# Endotoxin tolerance in mast cells, its consequences for IgE-mediated signalling, and the effects of BCL3 deficiency

**DOI:** 10.1038/s41598-017-04890-4

**Published:** 2017-07-03

**Authors:** Magdalena Poplutz, Maryna Levikova, Juliane Lüscher-Firzlaff, Marina Lesina, Hana Algül, Bernhard Lüscher, Michael Huber

**Affiliations:** 1 Institute of Biochemistry and Molecular Immunology, Medical School, RWTH Aachen University, Aachen, Germany; 2 Institute of Biochemistry and Molecular Biology, Medical School, RWTH Aachen University, Aachen, Germany; 3 Molecular Gastroenterology, Medical Clinic II, University Hospital Klinikum Rechts der Isar, TU Munich, Munich, Germany; 4 Institute of Molecular Cancer Research, University of Zürich, Zürich, Switzerland

## Abstract

Stimulation with lipopolysaccharide (LPS; endotoxin) not only causes rapid production of proinflammatory cytokines, but also induces a state of LPS hypo-responsiveness to a second LPS stimulation (endotoxin tolerance (ET)). Murine bone marrow-derived MCs (BMMCs) and peritoneal MCs (PMCs) developed ET as shown by an abrogated production of *Il6*/*Tnf* RNAs and IL-6/TNF-α proteins. In naive BMMCs, LPS stimulation induced a transient decline in the trimethylation of lysine 9 of the core histone H3 (H3K9me3), a suppressive chromatin mark, at the *Il6*/*Tnf* promoters, which correlated with p50(NFκB) and p65(NFκB) binding. Both demethylation and NFκB binding were abrogated in tolerant cells. In addition, cytosolic NFκB activation was suppressed in tolerant BMMCs. Intriguingly, antigen stimulation of naive and tolerant MCs induced comparable production of *Il6/Tnf* and IL-6/TNF-α, although ET also affected antigen-triggered activation of NFκB; pharmacological analysis indicated the importance of Ca^2+^-dependent transcription in this respect. In macrophages, the IκB member BCL3 is induced by LPS and known to be involved in ET, which was not corroborated comparing wild-type and *Bcl3*-deficient BMMCs. Interestingly, *Bcl3*-deficient PMCs produce markedly increased amounts of IL-6/TNF-α after LPS stimulation. Collectively, ET in MCs is BCL3-independent, however, in PMCs, BCL3 negatively regulates immediate LPS-induced cytokine production and quantitatively affects ET.

## Introduction

Recognition of and reaction to bacteria represents a central task of the immune system. For the execution of this function, immune cells, in particular myeloid immune cells (including macrophages (MΦs) and mast cells (MCs)), are equipped with so-called pattern-recognition receptors, e.g. Toll-like receptors (TLRs). TLRs recognize pathogen-associated molecular patterns (PAMPs), such as lipopolysaccharide (endotoxin, LPS; recognition by TLR4) and lipopeptides (recognition by TLR1, TLR2, and TLR6), which are essential for microbial survival and hence allow successful mammalian immune defence by means of genetically encoded receptors and their downstream signalling response including, amongst others, the NFκB and MAPK pathways. A hallmark of this immune response is the production of different proinflammatory cytokines (e.g. IL-6 and TNF-α)^1^.

Stimulation of LPS-pretreated cells with a second dose of LPS results in an attenuated or even a completely abrogated proinflammatory response. This phenomenon is called endotoxin tolerance (ET) and represents a protective mechanism to prohibit overproduction of proinflammatory cytokines and to avoid tissue damage^2^. ET has been studied mainly in monocytes and MΦs. Various molecules/mechanisms have been suggested to contribute to the development of ET ranging from the down-regulation of activating components (e.g. TLR4 and IRAK1^3, 4^) to the induction of inhibitory proteins (such as IRAK-M, SOCS1, SHIP1, A20, and BCL3^5–8^). In most instances these proteins are involved in the regulation of NFκB activation at different steps of the pathway. Interestingly, in response to LPS, endotoxin-tolerant macrophages did not only show suppressed production of proinflammatory cytokines, but they additionally exerted increased expression of anti-inflammatory mediators and antimicrobial effectors^9, 10^. Hence, ET is more than inhibition of proinflammatory cytokine production and therefore has recently been referred to as “LPS-induced cellular reprogramming”^2^. From a mechanistic point of view, the efficiency of the transcriptional and translational processes as well as mRNA and protein stability is critical for attenuated or augmented expression of relevant genes and proteins in the development of ET. Particular importance has been assigned to epigenetic processes, including histone acetylation and methylation, to control the chromatin state and thus the expression of ET-associated genes^9, 11–13^.

MCs are primarily localized to tissues with environmental contact, such as skin and mucosal surfaces of the gut and the lung. Thus, MCs are likely to be amongst the first cells that encounter bacteria and/or bacterial constituents like LPS and lipopeptides. Unlike MΦs, MCs do not express mCD14 and thus recognize LPS via TLR4 in an mCD14-independent manner^14^. This affects the ability to recognize different chemotypes of LPS with MCs reacting very efficiently to rough forms of LPS (R-LPS)^14^. Furthermore, MCs are not equipped with a fully functional TRAM-TRIF pathway and thus LPS stimulation of MCs only results in MYD88-dependent production of proinflammatory cytokines, but not in expression of type I interferons^15, 16^. It has been demonstrated that this lack of production of type I interferons allows for an efficient mobilization of neutrophils upon infection with *Listeria monocytogenes* ^15^.

In this report, we describe induction of ET by R-LPS in murine bone marrow-derived MCs (BMMCs) and peritoneal MCs (PMCs). LPS-induced production of proinflammatory cytokines was suppressed in tolerant cells and ET was accompanied by an inhibition of the NFκB and p38 signalling pathways as well as stabilization of repressive chromatin marks at target gene promoters. We further show that IgE-mediated proinflammatory cytokine expression in tolerant BMMCs was normal, although the down-regulation of NFκB and p38 pathways in tolerant cells was not compensated. To analyse the molecular basis of ET in MCs more closely, we concentrated on the IκB family member BCL3, the expression of which was upregulated in tolerant cells. No difference was observable between *Bcl3*−/− and wild-type (WT) BMMCs. In peritoneal MCs, however, TNF-α expression was markedly suppressed by BCL3 in both naive and tolerant cells. Hence, BCL3 controls ET in PMCs in a quantitative rather than qualitative manner.

## Results

### An LPS-induced dynamic change in H3K9 trimethylation is abrogated in tolerant mast cells

**Figure 1:**
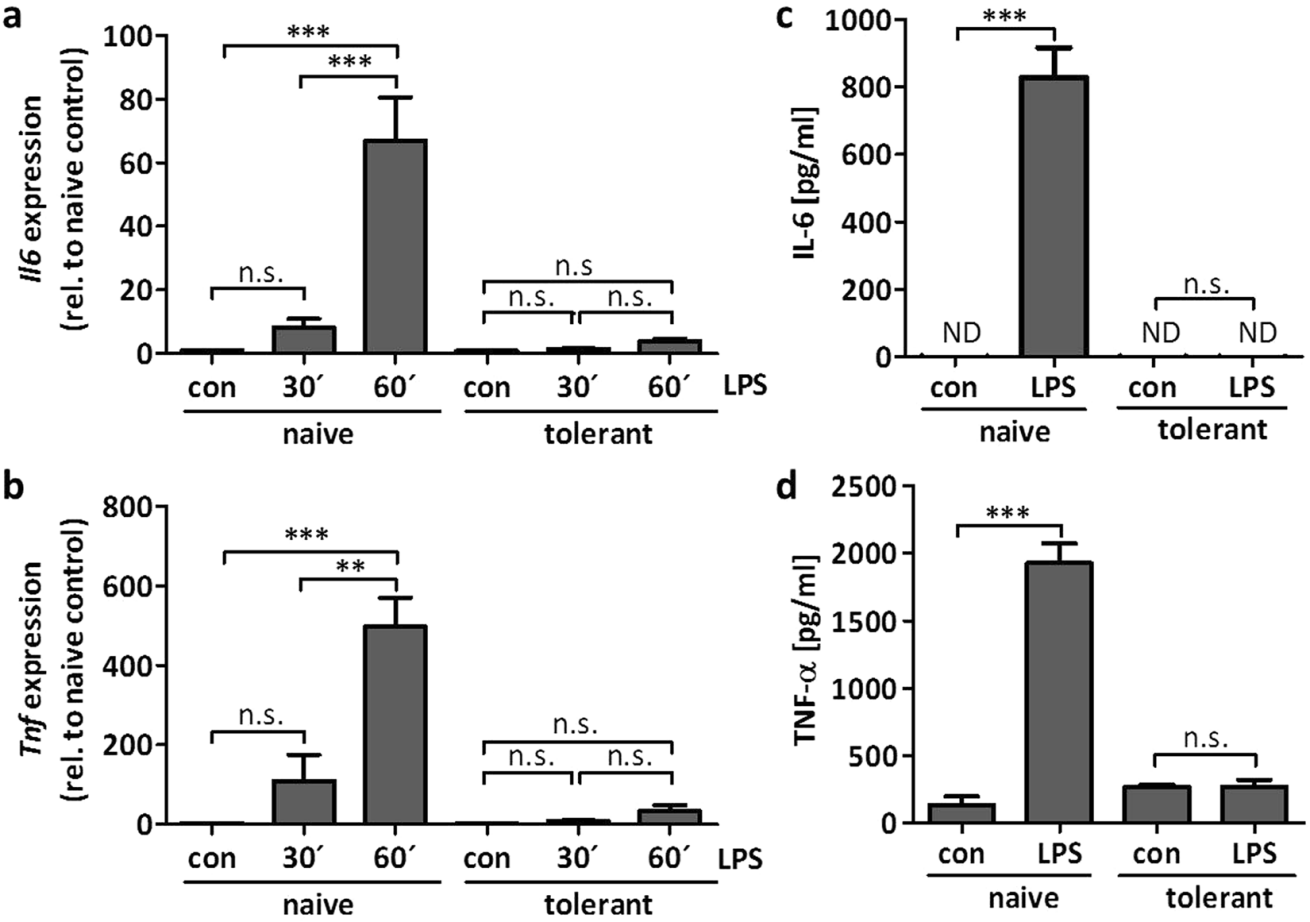
Murine BMMCs can develop ET. BMMCs were pretreated with LPS (300 ng/ml) for 24 h to induce ET. For analysis of the cytokine expression, the cells were stimulated with LPS (1 µg/ml) for 30 min and 60 min and the gene expression of *Il6* (**a**) and *Tnf* (**b**) was measured. Data show mean ± SD from n = 3 independent experiments. Two-sided ANOVA with Tukey HSD post-hoc test and one-sample *t*-Test were performed to calculate the p-values. Multiple testing p-values were corrected by FDR. Furthermore, LPS-pretreated and naive cells were stimulated with LPS (1 µg/ml) for 4 h to analyse protein production of IL-6 (**c**) and TNF-α (**d**). Data show mean ± SD from three independent experiments. Student’s *t*-Test was performed to calculate the p-values. **p < 0.01, ***p < 0.001.

Using BMMCs, it has been demonstrated that MCs can undergo ET^7, 17, 18^. Differentiation of BMMCs is a time-consuming process and their phenotype is known to be sensitive to the differentiation conditions used, such as the type of FCS and the source and composition of cytokines. Moreover, various types and sources of LPS are used by different laboratories. We aimed to connect our study to previous studies^7, 17, 18^ and thus, we first verified that the BMMCs in use can become tolerant in response to an initial stimulation with LPS (R595 from *Salmonella minnesota*). BMMCs were left untreated (“naive”) or pretreated with LPS (300 ng/ml) for 24 h (“tolerant”). Subsequently, cells were stimulated with LPS (1 µg/ml) and production of *Il6* and *Tnf* mRNA as well as IL-6 and TNF-α protein was measured by RT-qPCR and ELISA, respectively. Indeed, compared to naive BMMCs *Il6/Tnf* and IL-6/TNF-α production was significantly suppressed in tolerant cells in response to LPS (Fig. 1), verifying the applicability of the used cellular model and stimulus to study mechanisms of ET in MCs.

**Figure 2:**
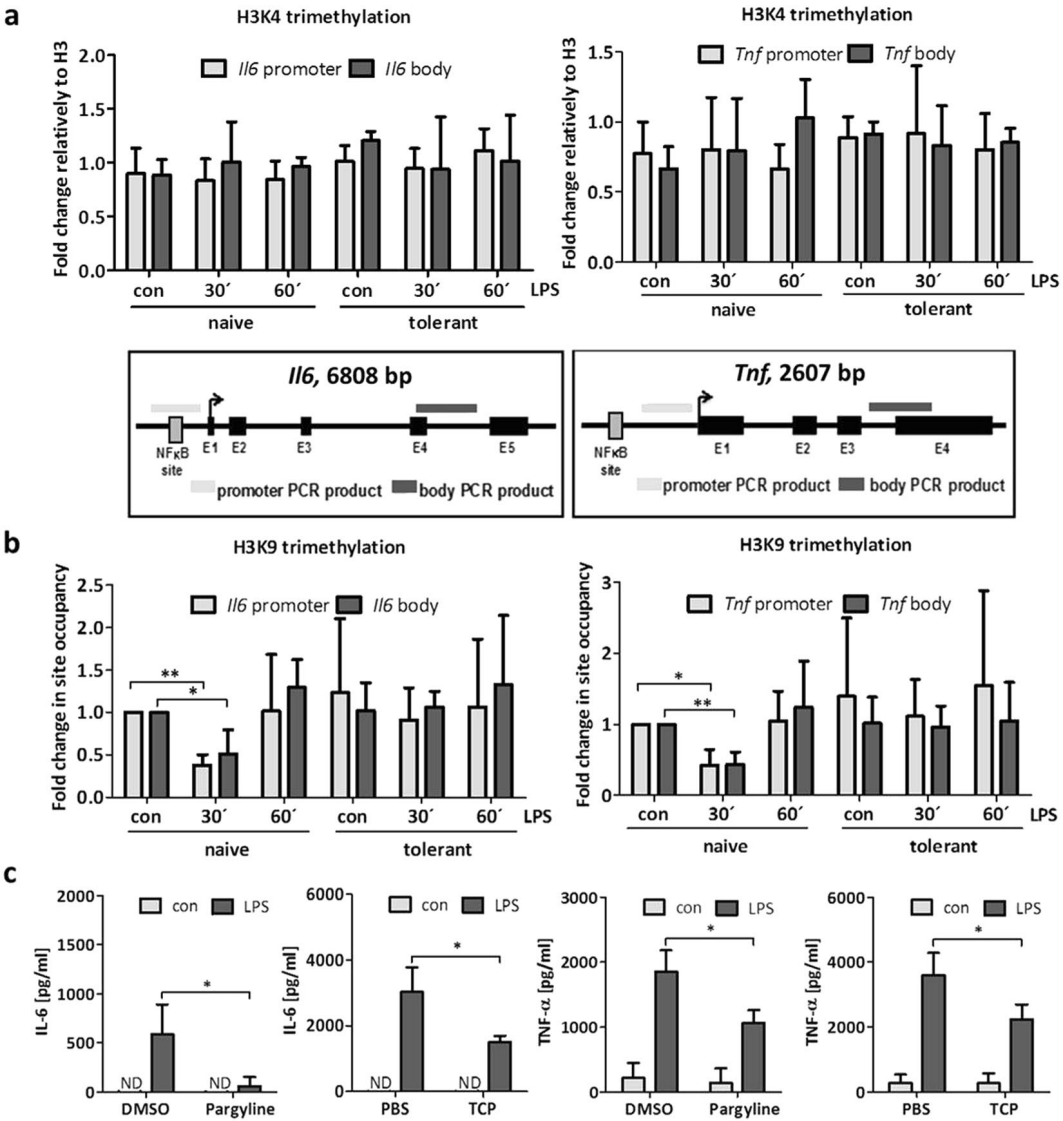
Histone H3K9 trimethylation is reduced in stimulated naive BMMCs and inhibition of histone demethylation leads to reduced cytokine production. BMMCs were pretreated with LPS (300 ng/ml) for 24 h to induce ET. To analyse histone modifications in naive and tolerant cells, the cells were stimulated with LPS (1 µg/ml) for 30 min and 60 min and ChIP analysis was performed. Fold change in site occupancy of H3K4me3 (**a**) and H3K9me3 (**b**) at the *Il6* and *Tnf* gene was measured. Data show mean ± SD from n = 3 independent experiments. Two-sided ANOVA with Tukey HSD post-hoc test and one-sample *t*-Test were performed to calculate the p-values. Multiple testing p-values were corrected by FDR. Localisation of PCR products within the *Il6* gene and the *Tnf* gene are depicted. Furthermore, cells were incubated with the LSD1 inhibitors Pargyline (1000 µM) or TCP (250 µM) for 2 h and stimulated with LPS (2 µg/ml) for 4 h to analyse protein production of IL-6 and TNF-α (**c**). Data show mean ± SD from n = 3 independent experiments with three biological samples. Student’s *t*-Test was performed to calculate the p-values. *p < 0.05, **p < 0.01, ***p < 0.001.

In MΦs, ET appears to be associated with transient silencing of *Il6* by means of epigenetic histone modifications^9^. LPS-stimulated naive MΦs showed increased H3K4 trimethylation (H3K4me3) at the *Il6* promoter, which was significantly reduced in LPS-triggered tolerant MΦs^9^. Interestingly, neither in naive nor in tolerant BMMCs did LPS stimulation cause any significant alteration in H3K4me3 at the *Il6* and *Tnf* promoters as well as in the gene bodies (Fig. 2a). Whereas H3K4me3 is considered an activating modification, present at promoters of both actively transcribed and poised genes, H3K9me3 appears to be a more stable suppressive mark^19^, changes of which have not been observed in tolerant MΦs^9^. In BMMCs we found that at both the *Il6* and *Tnf* promoters as well as in the gene bodies H3K9me3 was significantly and transiently reduced in LPS-stimulated naive BMMCs, whereas no such change was measurable in tolerant cells in response to LPS (Fig. 2b). This suggested that in tolerant LPS-stimulated BMMCs, in contrast to naive BMMCs, basal repression was sustained. Indeed, inhibition of the H3K9 demethylase LSD1 by the pharmacological LSD1-specific inhibitors pargyline and TCP^20, 21^ resulted in significant reduction of LPS-induced IL-6 and TNF-α production in naive BMMCs (Fig. 2c), indicating that H3K9me3 demethylation represents a crucial step in the process of LPS-induced production of proinflammatory cytokines.

### Binding of NFκB to *Il6* and *Tnf* promoters is restrained in tolerant mast cells

**Figure 3:**
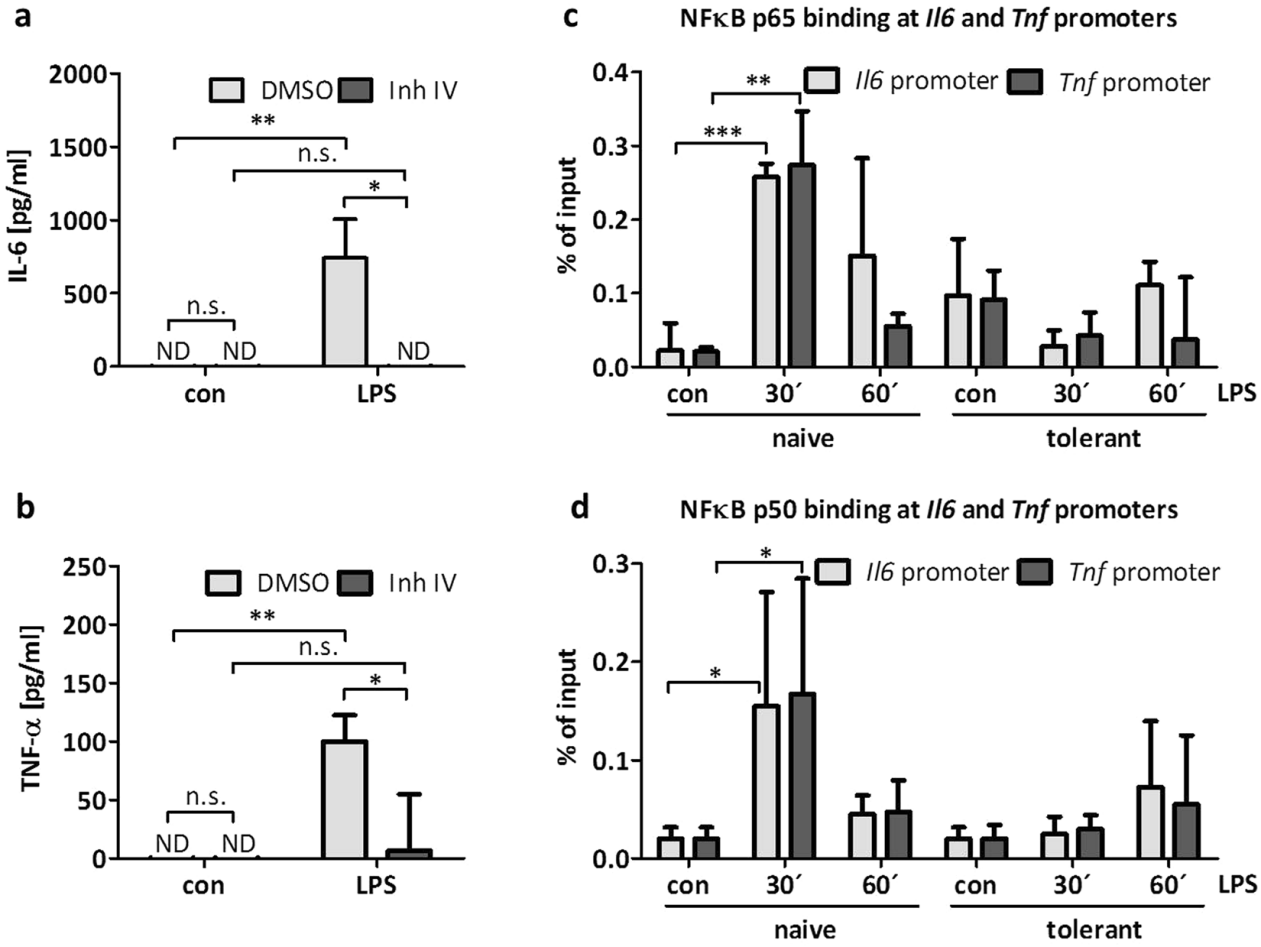
NFκB p65 and p50 binding to cytokine promoters is reduced in tolerant BMMCs. BMMCs were treated with the IKK-β inhibitor Inh IV (3 µM) for 1 h and then stimulated with LPS (300 ng/ml) for 4 h to measure IL-6 (**a**) and TNF-α (**b**) protein production. Data show mean ± SD from n = 3 independent experiments. Student’s *t*-Test was performed to calculate the p-values. To induce ET, BMMCs were incubated with LPS (300 ng/ml) for 24 h. Cells were left unstimulated (con) or stimulated with LPS (1 µg/ml) for 30 min and 60 min and ChIP analysis was performed to measure NFκB p65 (**c**) and NFκB p50 (**d**) binding to *Il6* and *Tnf* promoters. Data show mean ± SD from three independent experiments. Two-sided ANOVA with Tukey HSD post-hoc test was performed to calculate the p-values. Multiple testing p-values were corrected by FDR. *p < 0.05, **p < 0.01, ***p < 0.001.

Our data so far suggest that a reduction in H3K9me3 at the *Il6* and *Tnf* promoters correlates with enhanced expression in response to LPS, which did not occur in tolerant cells. LPS-induced production of IL-6 and TNF-α is dominantly dependent on the NFκB pathway, as could be corroborated by a marked decline of IL-6 and TNF-α production in the presence of the pharmacological IKK2 (IKK-β) inhibitor, inhibitor IV (Fig. 3a and b). Under these conditions, inhibitor IV treatment did not affect cell viability (data not shown). Thus, the NFκB proteins, p50 and p65, were expected to bind to the *Il6* and *Tnf* promoters in naive LPS-stimulated BMMCs, but not in LPS-treated tolerant cells. This was analysed by ChIP assays using p50- and p65-specific antibodies and measuring binding of p50 and p65 to the *Il6* and *Tnf* promoters. Indeed, correlating with the observed kinetics of H3K9me3 de- and remethylation (Fig. 2), p50 and p65 bound to *Il6* and *Tnf* promoters in LPS-stimulated naive, but not in LPS-treated tolerant BMMCs (Fig. 3c and d). This suggested that the recruitment of NFκB p50 and p65 and the changes in H3K9 trimethylation are interconnected in response to LPS.

### Compromised activation of the NFκB pathway in LPS-tolerant BMMCs

**Figure 4:**
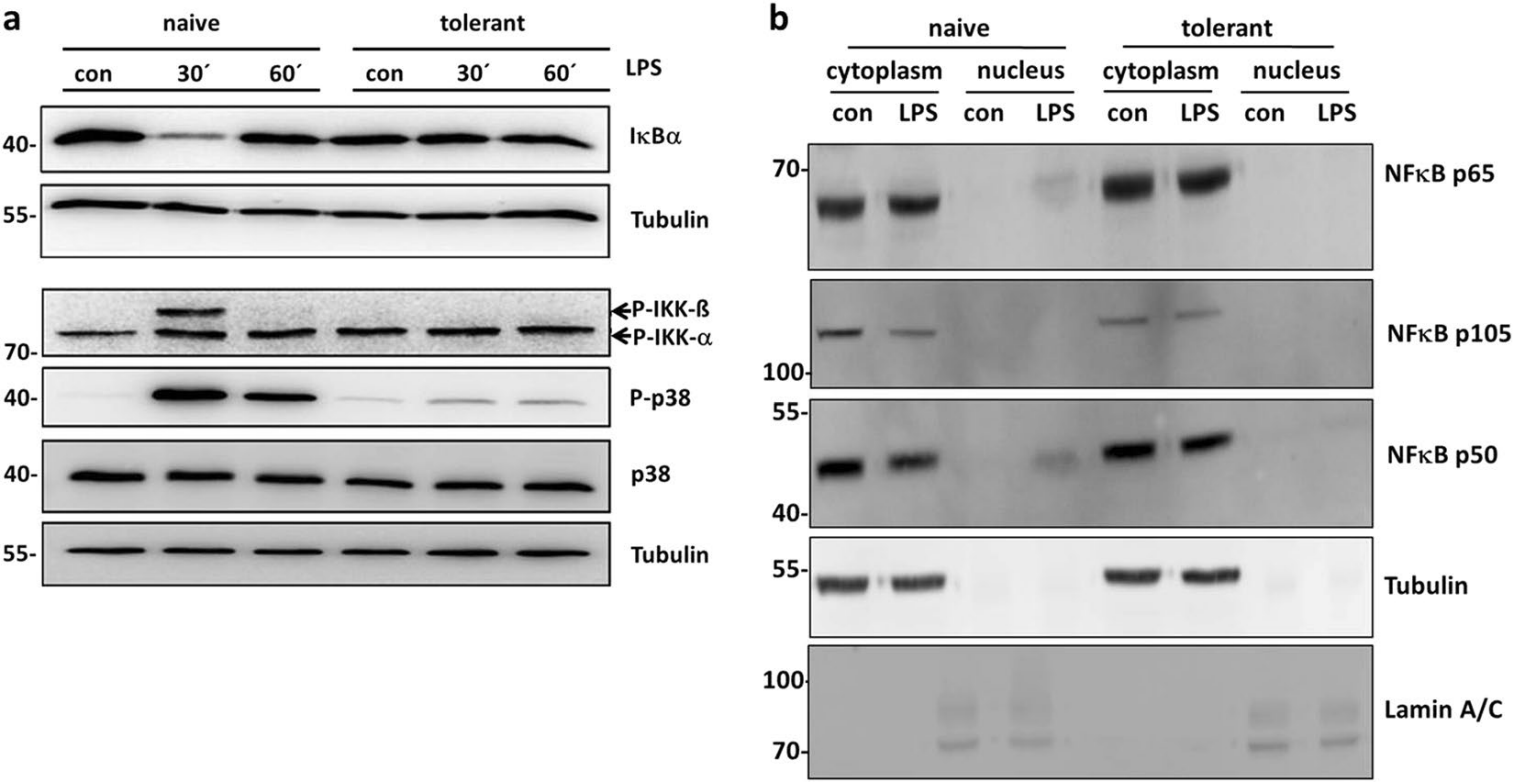
Impaired TLR4 signalling in tolerant BMMCs. Cells were preincubated with LPS (300 ng/ml) for 24 h to induce ET and stimulated with LPS (1 µg/ml) for 30 min and 60 min. Subsequently, cells were lysed in the presence of phosphatase inhibitors. SDS-PAGE and Western blotting with antibodies specific for IκBα, P-IKK-α/-β, P-p38, p38 and Tubulin (loading control) was performed (**a**). Because proteins of comparable size (p38 and IκBα) were analysed, the same lysates were separated on two gels with an anti-Tubulin loading control on each gel. Moreover, naive and tolerant cells were stimulated with LPS (2 µg/ml) for 60 min and a cytosol/nucleus fractionation was performed, followed by subsequent SDS-PAGE and Western blotting with antibodies specific for NFκB p65, NFκB p105 and p50 (both are detected by the same antibody), Tubulin, and Lamin A/C as loading controls (**b**). Western Blots are representative of two independent experiments with two biological samples.

The inability to measure interaction of p50/p65 with the *Il6* and *Tnf* promoters in tolerant BMMCs might be due to the stable presence of closed chromatin (Fig. 2), the absence of activation of the NFκB pathway or both. Activation of NFκB includes cytoplasmic phosphorylation as well as degradation of the inhibitory protein IκBα and subsequent nuclear translocation of p50 and p65. Naive and tolerant BMMCs were stimulated with LPS and phosphorylation/activation of IKK-α/-β as well as expression of IκBα were analysed by Western blotting. Whereas IKK-β was phosphorylated and IκBα was degraded in LPS-stimulated naive cells, these changes were not detectable in tolerant BMMCs in response to LPS (Fig. 4a). Fitting to the kinase TAK1 being a common signalling element upstream of IKK and p38^MAPK^ activation^22^, LPS-induced p38 phosphorylation was severely reduced in tolerant compared to naive cells (Fig. 4a). These results suggested that in tolerant cells, p50 and p65 were not released from IκBα and hence, did not translocate into the nucleus. Thus, the unavailability of p50 and p65 in the nucleus might explain the lack of H3K9me3 reduction at the *Il6* and *Tnf* promoters and consequently the restricted expression of proinflammatory cytokines in tolerant BMMCs.

To address the latter, unstimulated and LPS-stimulated naive as well as tolerant BMMCs were separated into cytoplasmic and nuclear fractions and presence of p65 and p50 was analysed by immunoblotting. Successful separation was verified by blotting against tubulin (cytoplasm) and lamin A/C (nucleus). Both p65 and p50 were detectable in the nuclear fraction of LPS-stimulated naive BMMCs (Fig. 4b), corroborating cytoplasmic IKK-driven degradation of IκBα. As expected from our analysis of NFκB pathway activation in tolerant cells, p65 and p50 were not detected in the nuclear fractions of both unstimulated and LPS-stimulated tolerant BMMCs (Fig. 4b). Moreover, the cytosolic precursor of p50, p105, which was also detected by the anti-p50 antibody, was markedly reduced in LPS-stimulated naive cells (Fig. 4b). In conclusion, LPS-tolerant BMMCs exerted suppressed activation of the cytosolic TAK1/NFκB pathway in response to LPS stimulation. This resulted in marginal to no translocation of p50 and p65 into the nucleus as well as poor interaction of these transcription factors with proinflammatory cytokine promoters (*Il6*/*Tnf*). This appeared to correlate or even is the reason for loss of reduction in H3K9 trimethylation at these promoters.

### A TLR4-independent stimulus induces transcription of proinflammatory genes in tolerant BMMCs despite enduring suppression of NFκB activation

**Figure 5:**
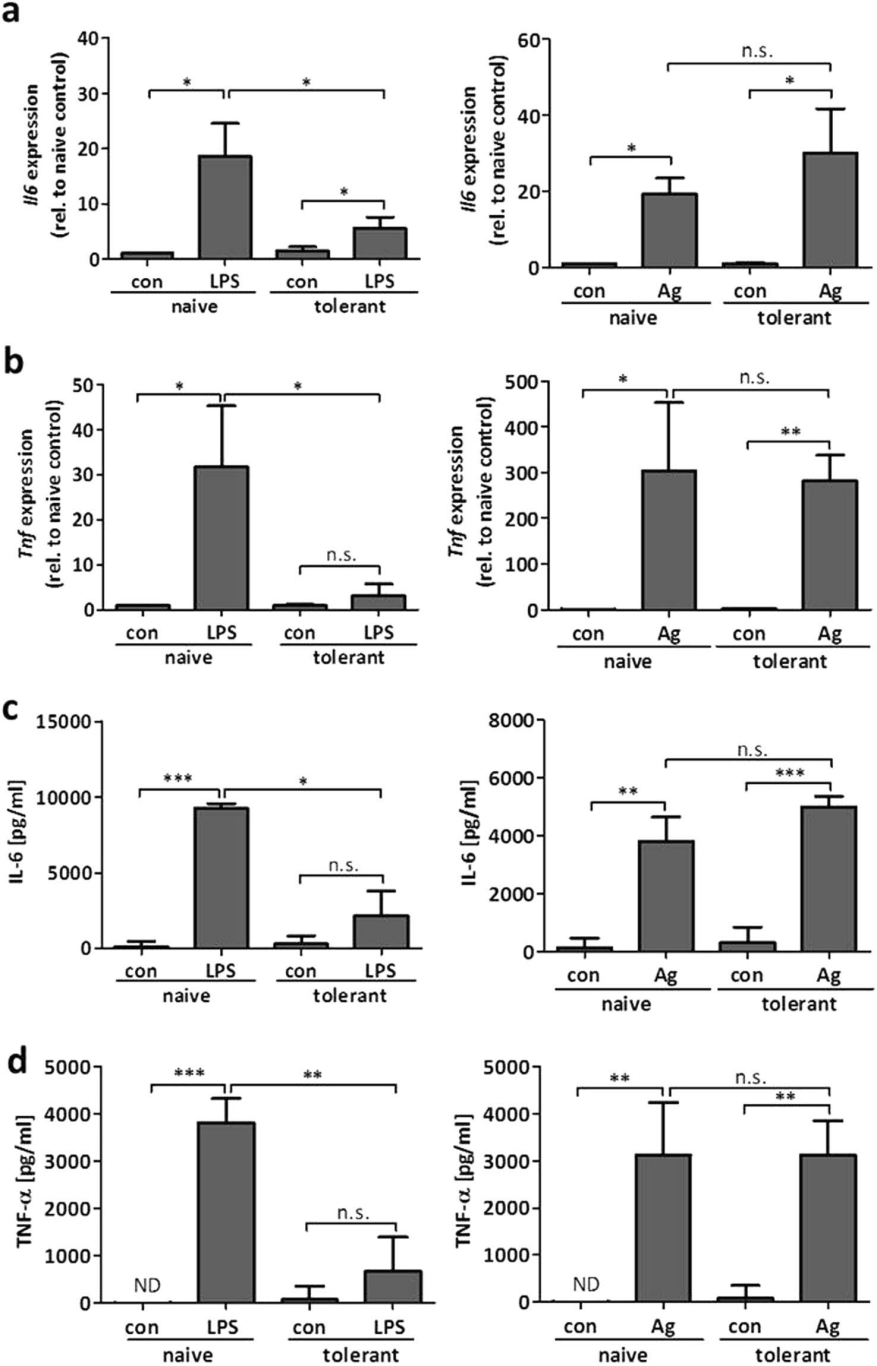
Antigen-triggered cytokine expression is not affected in tolerant BMMCs. To induce ET, BMMCs were treated with LPS (300 ng/ml) for 24 h and loaded with IgE (150 ng/ml) overnight. Tolerant and naive cells were stimulated with LPS (1 µg/ml) or Ag (DNP-HSA; 20 ng/ml) for 90 min and *Il6* (**a**) and *Tnf* (**b**) gene expression was measured. Data show mean ± SD from three independent experiments. Student’s *t*-Test and one-sample *t*-Test were performed to calculate the p-values. Multiple testing p-values were corrected by FDR. Furthermore, naive and tolerant cells were incubated with LPS (1 µg/ml) or Ag (DNP-HSA; 20 ng/ml) for 4 h to analyse protein production of IL-6 (**c**) and TNF-α (**d**). Data show mean ± SD from n = 3 independent experiments. Student’s *t*-Test was performed to calculate the p-values. *p < 0.05, **p < 0.01, ***p < 0.001.

We next asked if the lack of LPS-induced *Il6* and *Tnf* transcription in tolerant BMMCs can be overcome by stimulation with a TLR4-independent ligand, such as antigen (Ag), which stimulates MCs via the IgE-bound FcεRI. Compared to TLR4, the FcεRI couples to IKK by a different initial mechanism involving Bcl10 and MALT1^23^. BMMCs were left untreated or were pretreated with LPS for 24 h and concomitantly preloaded with DNP-specific IgE. Subsequently, naive and tolerant BMMCs were stimulated with LPS or Ag (DNP-HSA) for 90 min and *Il6* and *Tnf* mRNAs were measured by RT-qPCR. Whereas production of *Il6/Tnf* mRNA was severely suppressed in LPS-stimulated tolerant BMMCs, there was no significant change in *Il6/Tnf* transcription between naive and tolerant BMMCs in response to Ag (Fig. 5a and b). In agreement, production and secretion of IL-6 and TNF-α were diminished in LPS-stimulated tolerant compared to naive cells. Ag-triggered IL-6 and TNF-α production, however, were comparable in naive and tolerant BMMCs (Fig. 5c and d).

**Figure 6:**
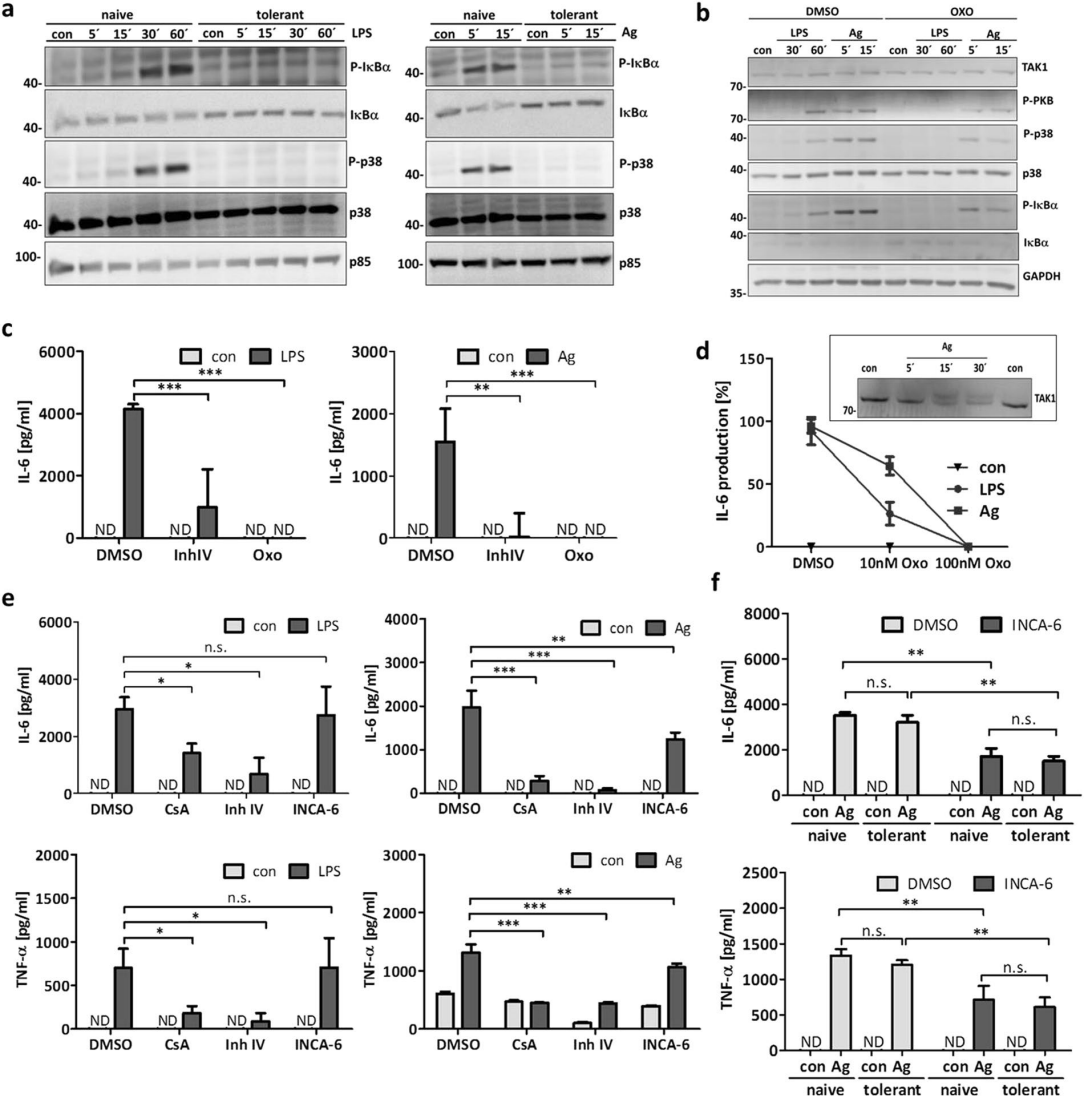
Differential activation of signalling pathways in tolerant cells in response to antigen stimulation. To induce ET, BMMCs were treated with LPS (300 ng/ml) for 24 h and loaded with IgE (150 ng/ml) overnight. Naive and tolerant cells were stimulated with LPS (1 µg/ml) or Ag (DNP-HSA; 20 ng/ml) for indicated times. Immunoblotting against P-IκBα, IκBα, P-p38, p38 and p85 (loading control) was performed (**a**). Additionally, cells were incubated with the TAK1 inhibitor (5Z)-7-Oxozeaenol (Oxo; 300 nM) for 30 min and stimulated with LPS or Ag for indicated times. Subsequently, Western blotting was performed with antibodies against TAK1, P-PKB, P-IκBα, IκBα, P-p38, p38 and GAPDH (loading control) (**b**). Western Blots are representative of three independent experiments. Furthermore, BMMCs were incubated with Oxo (300 nM) for 30 min and stimulated with LPS or Ag for 4 h to analyse IL-6 production (**c**). Data show mean ± SD from n = 3 independent experiments. Two-sided ANOVA with Dunnet’s post-hoc test was performed to calculate p-values. To analyse the differential effect of Oxo on IL-6 production, BMMCs were treated with 10 nM and 100 nM Oxo and then left unstimulated (con) or stimulated with LPS and Ag for 4 h (**d**). Data show mean ± range from n = 2 independent experiments each measured in triplicates. Moreover, IgE-preloaded BMMCs were left unstimulated (con) or stimulated with Ag for indicated times and TAK1 was detected by Western blotting; shown in the insert to (**d**). Furthermore, BMMCs were treated with Cyclosporin A (CsA; 0.1 µM), IKK-β inhibitor InhIV (3 µM), and INCA-6 (3 µM) for 1 h and then stimulated with LPS and Ag for 4 h to measure IL-6 and TNF-α secretion (**e**). Data show mean ± SD from n = 3 independent experiments. Two-sided ANOVA with Dunnet’s post-hoc test was performed to calculate p-values. Tolerant and naive BMMCs were incubated with INCA-6 (3 µM) for 1 h and stimulated with Ag for 4 h to measure IL-6 and TNF-α production (**f**). Data show mean ± SD from n = 3 independent experiments. Student’s *t*-Test was performed to calculate p-values. *p < 0.05, **p < 0.01, ***p < 0.001.

**Figure 7:**
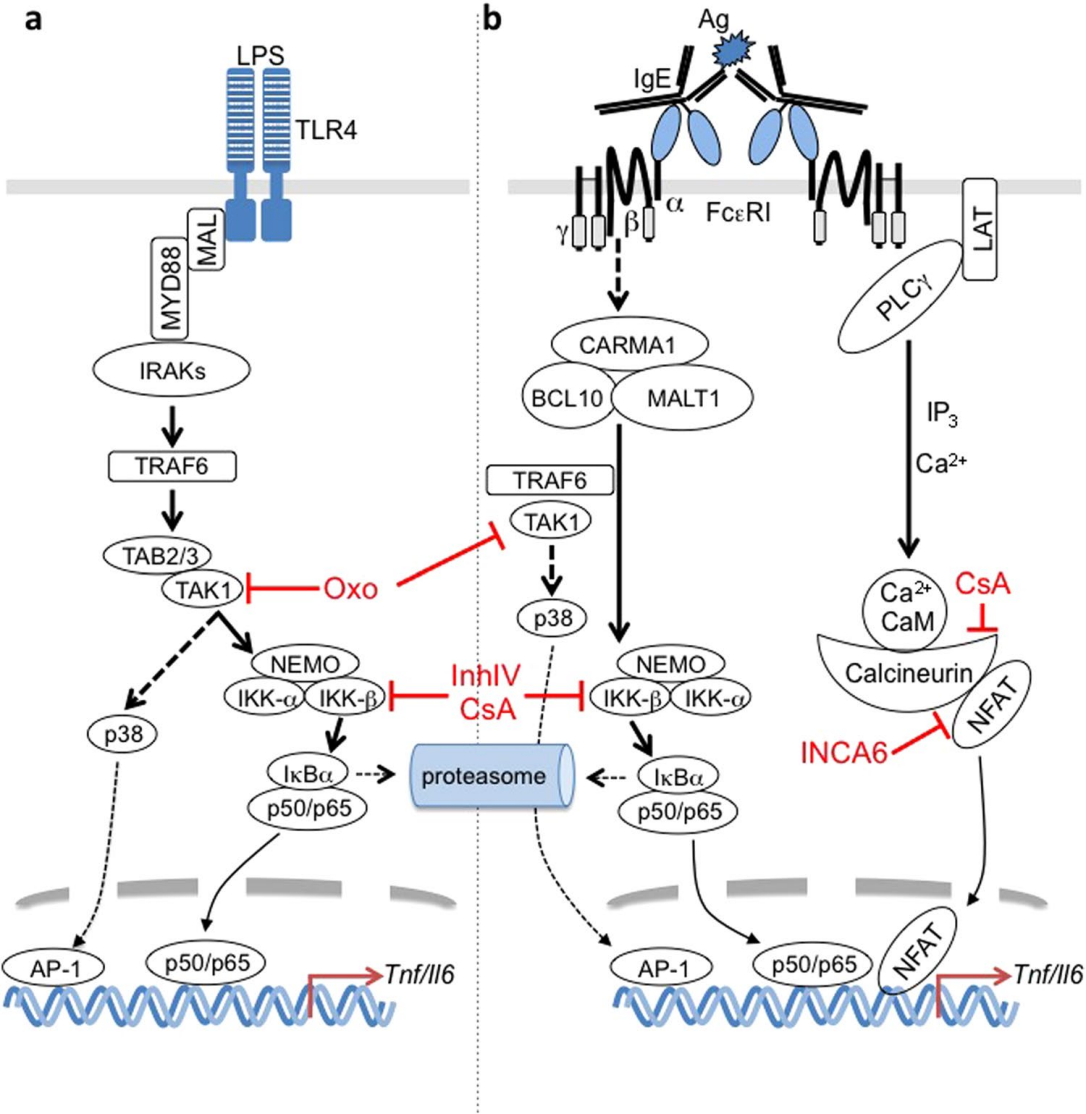
Differences and commonalities in TLR4- and FcεRI-activated production of proinflammatory cytokines. (**a**) LPS-induced stimulation of TLR4 in MCs results in activation of the MYD88 pathway. The E3 ligase TRAF6 mediates the activation of the Ser/Thr kinase TAK1, which allows for bifurcation of the signal into the NFκB and MAPK pathways leading to transcription of proinflammatory genes. This is suppressed by inhibition of TAK1 (by Oxo) and IKK-β (by InhIV; CsA inhibits calcineurin, indirectly contributing to suppression of IKK-β^31, 33^). (**b**) Ag-induced crosslinking of the IgE-bound FcεRI leads to NFκB activation mediated by the CARMA1/BCL10/MALT1 complex as well as TRAF6 and TAK1^23, 52^. In contrast to TLR4 signalling, FcεRI triggering leads to PLCγ/IP_3_-controlled store-operated Ca^2+^ influx allowing for dephosphorylation/activation of NFAT by Ca^2+^/CaM-activated calcineurin. Whereas CsA directly and indirectly inhibits calcineurin and IKK-β, respectively, INCA6 selectively blocks the interaction of calcineurin and NFAT^30^.

Considering the NFκB pathway as the central element for the induction of proinflammatory cytokines, these data suggested that Ag-triggered IκBα phosphorylation^24^ should be comparable in naive and tolerant BMMCs, whereas the LPS-induced reaction should be abrogated in tolerant cells. Unexpectedly, IκBα phosphorylation was suppressed in tolerant BMMCs in response to both LPS and Ag (Fig. 6a). In addition, LPS- and Ag-induced p38 phosphorylation was severely reduced in tolerant cells (Fig. 6a). This suggested participation of TAK1 in response to both TLR4- and FcεRI-mediated BMMC stimulation, because TAK1 plays a role upstream of both NFκB and p38 activation^22^ and both TLRs and antigen receptors appear to be able to signal via TAK1^25^ (Fig. 7).

Indeed, treatment of naive IgE-loaded BMMCs with the selective TAK1 inhibitor 5z 7-Oxozeaenol (Oxo)^26^ suppressed phosphorylation of IκBα and p38 in response to both LPS and Ag (Fig. 6b). However, whereas the LPS-induced responses were completely blocked by Oxo, Ag-triggered phosphorylation of IκBα and p38 were only partially suppressed by Oxo pretreatment (Fig. 6b). Moreover, PKB phosphorylation was strongly inhibited by Oxo in response to LPS, but appeared only partially inhibited after Ag triggering. Applying a commonly used concentration of Oxo (300 nM), both LPS and Ag-induced IL-6 production were completely inhibited (Fig. 6c). However, in agreement with the higher sensitivity for Oxo treatment in response to LPS (Fig. 6b), LPS-induced IL-6 production was more sensitive to Oxo-mediated TAK1 inhibition compared to the Ag-triggered response, as revealed by titration of Oxo (Fig. 6d). In agreement with TAK1 activation/phosphorylation in response to Ag, approximately 50% of the cellular TAK1 molecules showed a significant size shift (Fig. 6d, insert).

Normal Ag-triggered proinflammatory cytokine production in tolerant BMMCs, despite suppressed TAK1-mediated NFκB and p38 activation, suggested a compensatory role of (an) additional pathway(s) for the production of IL-6 and TNF-α. In this respect, Ag is known as a strong inducer of Ca^2+^ mobilisation and degranulation in BMMCs, whereas LPS is not^27, 28^ (Fig. 7). Because the Ca^2+^/calcineurin-dependent transcription factor NFAT has been demonstrated to be involved in Ag-triggered TNF-α production^29^, we analysed the effect of the selective, calcineurin/NFAT complex-disrupting drug INCA-6^30^ on LPS- and Ag-stimulated IL-6 and TNF-α production. Indeed, INCA-6 significantly reduced Ag-triggered IL-6 and TNF-α production, whereas it did not impact on LPS-induced production of these proinflammatory cytokines (Fig. 6e). As expected, INCA-6-mediated suppression of Ag-triggered IL-6 and TNF-α production was observed in both naive and tolerant BMMCs (Fig. 6f). The phosphatase calcineurin, the target of the well-established inhibitor cyclosporine A (CsA), was shown to be involved not only in NFAT dephosphorylation, but also, amongst others, in activation of the NFκB pathway^31–33^ (Fig. 7). In line, CsA reduced proinflammatory cytokine production in response to both LPS and Ag, however, the effect in Ag-triggered cells was markedly stronger (Fig. 6e). The IKK-β inhibitor IV, as expected, blocked the response to both stimuli (Fig. 6e). These results indicated that Ag is able to induce production of IL-6 and TNF-α in endotoxin-tolerant BMMCs in a Ca^2+^-dependent manner in the context of suppressed TAK1-p38/NFκB pathways.

### BCL3 deficiency does not alter the development of ET in BMMCs

**Figure 8:**
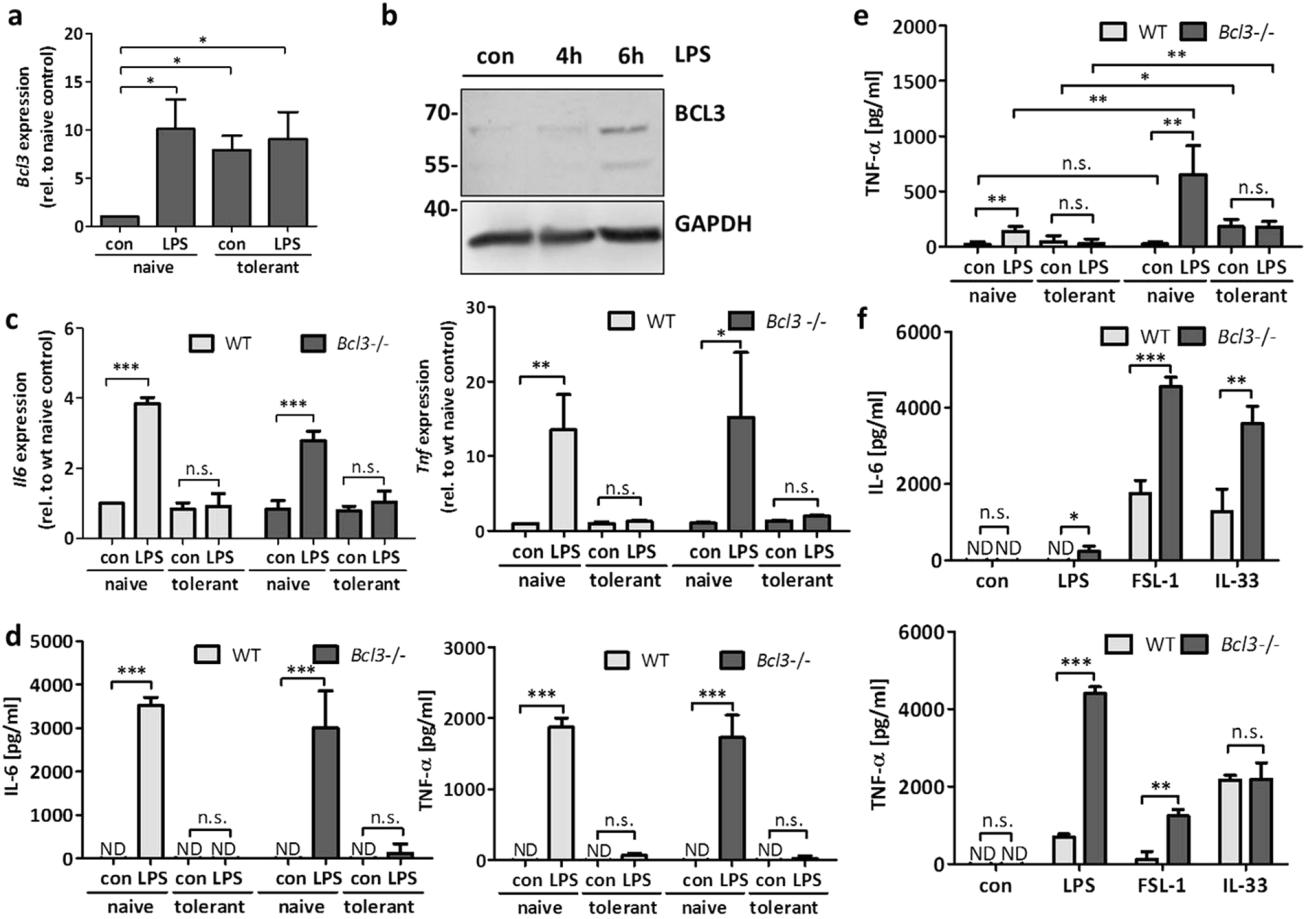
BCL3 controls cytokine production in PMCs without altering the quality of ET. BMMCs were left untreated or incubated with LPS (300 ng/ml) for 24 h to induce ET. Naive and tolerant BMMCs were then stimulated with LPS (1 µg/ml) for 90 min and *Bcl3* gene expression was analysed (**a**). Data show mean ± SD from n = 3 independent experiments. One-sample *t*-Test was performed to calculate the p-values. Moreover, cells were stimulated with LPS (1 µg/ml) for 4 h and 6 h and lysates were analysed by Western Blotting with antibodies specific for BCL3 and GAPDH (loading control) (**b**). The Western blot is representative of three independent experiments with three biological samples. Next, BMMCs of WT and *Bcl3*−/− mice were pretreated with LPS (300 ng/ml) for 24 h to induce ET. Then the cells were stimulated with LPS (1 µg/ml) for 90 min to measure *Il6* and *Tnf* mRNA expression (**c**), and for 4 h to detect IL-6 and TNF-α protein secretion (**d**). Also, WT and *Bcl3*−/− PMCs were incubated with LPS (300 ng/ml) for 24 h to induce ET, and then stimulated with LPS (1 µg/ml) for 4 h to analyse TNF-α protein production (**e**). Moreover, WT and *Bcl3*−/− PMCs were stimulated with LPS (1 µg/ml), FSL-1 (1 µg/ml), and IL-33 (2 ng/ml) for 4 h and IL-6 and TNF-α protein secretion was measured (**f**). Data show mean ± SD from three independent experiments. Student’s *t*-Test and one-sample *t*-Test were performed to calculate the p-values. Multiple testing p-values were corrected by FDR. *p < 0.05, **p < 0.01, ***p < 0.001.

Several signalling proteins show altered expression in tolerant compared to naive cells. This can be the result of degradation/inhibition of positive regulators, such as MYD88 and IRAK1^3, 4^, or induction/activation of inhibitory signalling elements, such as IRAK-M, SOCS-1, SHIP1, and BCL3^5–8^. The fact that different proteins appear to be involved in the regulation of ET is in line with its important task to prevent chronic and devastating inflammation. Performing a preliminary Affymetrix transcriptome analysis of unstimulated and LPS-stimulated naive as well as tolerant BMMCs, mRNA of the IκBα family member BCL3 was found to be upregulated in LPS-stimulated naive as well as in unstimulated and stimulated tolerant cells (but no further induction in tolerant cells, i.e. it remains high in response to the first LPS signal), whereas this was not the case for MYD88, IRAK1, IRAK-M, SOCS-1, and SHIP1. This pattern for *Bcl3* was verified subsequently by RT-qPCR (Fig. 8a). In agreement, significantly enhanced expression of BCL3 protein was observed starting 6 h after primary LPS stimulation (Fig. 8b). We decided to analyse the role of BCL3 with respect to ET in MCs, since BCL3-deficient mice as well as BCL3-deficient MΦs have been shown previously to be unable to mount ET^8^. Hence, we generated and analysed BMMCs from WT and *Bcl3*−/− mice, which differentiated comparably as shown by expression of typical surface receptors (FcεRI and KIT) (Suppl. Fig. 1). Next the cells were left untreated or were treated with a first dose of LPS for 24 h and subsequently the resulting naive and tolerant cells were left without stimulus or stimulated with LPS and production of *Il6* and *Tnf* mRNA as well as IL-6 and TNF-α protein was measured. Unexpectedly, WT and *Bcl3*−/− BMMCs produced comparable amounts of *Il6*/*Tnf* and IL-6/TNF-α in naive or tolerant cells (Fig. 8c and d). These data showed that despite its upregulation during tolerance, BCL3 appears not to be involved in the regulation of LPS-induced proinflammatory cytokine production in BMMCs.

### BCL3 controls production of proinflammatory cytokines in naive and tolerant peritoneal mast cells

Murine MCs can be roughly classified into mucosal-type and connective tissue-type MCs. In contrast to BMMCs, PMCs belong to the connective tissue type. PMCs have been shown to exert a different phenotype compared to BMMCs, in particular with respect to the combination of proteases expressed in secretory lysosomes^34^. It is very likely that differences in protein expression between BMMCs and PMCs are not constrained to proteases and hence the induction of ET was analysed in PMCs by measuring TNF-α production. Indeed, PMCs became endotoxin tolerant in response to prolonged treatment with low levels of LPS. Compared to naive PMCs, tolerant PMCs did hardly produce TNF-α protein in response to subsequent LPS stimulation (Fig. 8e). The comparison to PMCs from BCL3-deficient mice revealed that *Bcl3*−/− PMCs did also develop ET (Fig. 8e). However, TNF-α production in unstimulated and LPS-stimulated tolerant *Bcl3*−/− PMCs was significantly increased compared to unstimulated naive *Bcl3*−/− PMCs (Fig. 8e). This suggested that BCL3 is required for the effective turning-off of TNF-α expression in response to LPS treatment. In support, LPS-induced production of TNF-α was markedly enhanced in naive *Bcl3*−/− PMCs in comparison to naive WT PMCs (Fig. 8e). Finally, to address the question if other TLRs or TLR-like receptors also are controlled by BCL3 regarding the induction of a proinflammatory response, WT and *Bcl3*−/− PMCs were stimulated with LPS, the TLR2/6 ligand FSL-1, and the IL-33 receptor ligand IL-33. Interestingly, whereas LPS and the lipopeptide FSL-1 induced a stronger TNF-α production in *Bcl3*−/− PMCs, there was no difference in response to IL-33 (Fig. 8f). However, *Bcl3*−/− BMMCs produced significantly higher amounts of IL-6 compared to WT cells in response to each stimulus (Fig. 8f). The difference in BCL3 dependence of IL-33-induced TNF-α and IL-6 production is not clear at the moment. In conclusion, these data indicated that the IκBα family member BCL3 does not qualitatively control ET in murine BMMCs and PMCs. However, in a MC type-specific manner, BCL3 restricts production of TNF-α and IL-6 in naive cells in response to ligands for TIR domain-containing receptors.

## Discussion

MCs are located at the interphase between self and environment and hence are in constant contact with pathogens and/or commensals. To avoid exaggerated proinflammatory activation of MCs by microbial constituents, such as LPS and lipopeptides, acquiring a state of tolerance should be beneficial. It has been demonstrated previously using BMMCs and a MC line that MCs can undergo ET^7, 17, 18^. Moreover, isolated human gut MCs express TLR4, but did not respond to LPS indicating tolerance^35^. In the present work, we verified LPS tolerance in BMMCs with respect to the secretion of IL-6 and TNF-α and extended this analysis to the epigenetic, transcriptional, and signalling levels.

An impressive wealth of data concerning regulation and effects of ET has been generated using monocytes and MΦs, which, however, cannot serve as a blueprint for MCs, since substantial differences in LPS signalling have been demonstrated between these cells. In contrast to MΦs, MCs do not express mCD14, they are not equipped with an efficient TRIF pathway and thus do not express IFN-α/β in response to LPS, and the PI3K pathway was shown to play an opposing role^14–16, 36, 37^.

A further difference was observed in the epigenetic regulation of LPS-induced *Il6* transcription. Whereas H3K4me3 was increased at the *Il6* gene in LPS-stimulated naive murine bone marrow-derived MΦs (which was then repressed in tolerant cells)^9^, no significant changes could be observed at the promoters and gene bodies of *Il6* and *Tnf* in LPS-treated BMMCs. Instead and in contrast to MΦs, both *Il6* and *Tnf* showed a transient decline in H3K9me3 at their promoters and in their gene bodies in BMMCs, which strongly correlated with recruitment of p50(NFκB) and p65(NFκB). Changes in H3K9me3 and p50/p65 recruitment were abolished in tolerant BMMCs. In line, pharmacological inhibition of respective demethylases attenuated LPS-induced proinflammatory cytokine production.

H3K9me3 is mostly discussed in the context of heterochromatin formation and long-term transcriptional repression. Reduction in H3K9me3 at proinflammatory genes in response to LPS has been reported in human monocyte-derived dendritic cells^38^. These authors proposed that demethylation of H3K9me3 generates a window of time, in which transcription of tightly controlled inducible genes is permitted. Accordingly, it was found in LPS-tolerant THP-1 cells, a human promonocytic cell line frequently used for investigations of ET, that H3K9 and DNA methylation interact to silence *TNF* expression in tolerant cells^13^. In tolerant cells, the *TNF* promoter is bound by the H3K9 methyltransferase G9a and the methylated histone serves as an interaction platform for the heterochromatin binding protein 1, which in turn recruits a DNA methyltransferase^13^. Moreover, the NFκB family member RELB was shown to interact with G9a and, together with HMGB1 and histone H1, to be crucial for epigenetic silencing^11, 12^. Interestingly, a preliminary transcriptome analysis of LPS-stimulated naive and tolerant BMMCs revealed increased transcription of *Relb* in unstimulated and LPS-stimulated tolerant BMMCs compared to naive cells (data not shown) and thus, the epigenetic tolerance mechanisms might possess significant similarities between human THP-1 cells and murine BMMCs.

Loss of NFκB activation in tolerant cells by different mechanisms has been reported (e.g. upregulation of the deubiquitinase A20 or degradation of IRAK1^18, 39, 40^), which could also be a reason for loss of NFκB binding in tolerant cells. Indeed, early phosphorylation and degradation of IκBα was suppressed in tolerant BMMCs. In addition, phosphorylation of p38 was strongly attenuated, suggesting that already at the level of the TAK1-containing signalling hub, activation is compromised in LPS-tolerant BMMCs. Moreover, consistent with previous work^18^, our preliminary experiments also showed IRAK1 degradation in response to the initial stimulation by LPS.

This suggests that loss of H3K9me3 demethylation in LPS-stimulated tolerant cells is taking place in addition to suppression of activation of initial cytosolic signalling pathways. This also fits with the idea that ET is a crucial protective mechanism, which has to be controlled by different means on various signalling levels to grant its functionality. This is supported by our observation that p50/p65 are translocated into the nucleus in LPS-stimulated naive BMMCs, but not in tolerant cells, indicating crosstalk of cytosolic and nuclear regulatory steps in the course of ET.

LPS-tolerant MCs exert various alterations in TLR4-mediated cytoplasmic and nuclear signals and it can be assumed that the tolerant behaviour expands to other ligand-receptor systems, which depend on closely related molecular mechanisms. Indeed, LPS-tolerant MCs have been demonstrated to be tolerant to TLR2 ligands (lipopeptides) as well as IL-33 (^18^ and our unpublished data). With respect to receptors that use significantly different activation mechanisms, such as the FcεRI, it has been shown that Ag-triggered secretion of IL-6 in tolerant BMMCs was not reduced compared to naive cells^17, 18^. We did corroborate and extend this by demonstrating that both *Il6/Tnf* and IL-6/TNF-α production in response to Ag were comparable in naive and tolerant BMMCs. In light of this observation, it was unexpected that the activation of the NFκB and p38 pathways was markedly suppressed in tolerant Ag-stimulated BMMCs compared to naive cells. This suggested that TAK1, which is known to be upstream of the NFκB-activating IKK complex as well as p38 in LPS-stimulated cells^22^, acts as a mediator in FcεRI signalling. Moreover, TAK1 has been demonstrated to be affected in LPS-tolerant macrophages^40^. In line, a selective TAK1 inhibitor, Oxo^26^, blocked both LPS- and Ag-induced proinflammatory cytokine production, however, with the LPS response being more sensitive than the Ag response. In agreement, IκBα and p38 phosphorylation were attenuated by TAK1 inhibition in response to both LPS and Ag stimulation. Interestingly, PKB phosphorylation was only affected by TAK1 inhibition in LPS-stimulated, but not Ag-triggered BMMCs. The reason for this is not clear at present. However, it offers a possible explanation for the seemingly normal cytokine production in Ag-stimulated tolerant BMMCs. The results of our pharmacological analysis points to a further reason for the differences in LPS- and Ag-induced proinflammatory cytokine production in LPS-tolerant BMMCs. In contrast to the LPS response, Ag-triggered cytokine production was attenuated by INCA-6, an inhibitor of the calcineurin-NFAT complex^30^, corroborating the importance of NFAT for Ag-triggered cytokine production^29^. This was in agreement with the inability of LPS to induce a Ca^2+^ response in MCs (own unpublished data). Calcineurin has been published to act on more targets than NFAT and to be involved in the control of the NFκB pathway^31–33^; thus, it was not surprising that compared to INCA-6, CsA suppressed both LPS- and Ag-induced cytokine generation. In conclusion, our results suggest that MCs use different transcription factors and respective response elements depending on the type of stimulus and thus, despite reduced NFκB and p38 activation in tolerant cells, Ag is able to induce proinflammatory cytokine production via a Ca^2+^-dependent pathway (Fig. 7). Our results further suggest that depending on the type of stimulus and signalling pathways used, tolerant MCs show changes in their response to TLR-independent stimuli due to the tolerance-induced alterations in diverse signalling networks. Autocrine mechanisms might be affected as well. To understand the effects of ET on MC activation by non-TLR ligands will be the aim of future studies.

In MΦs, BCL3 has been demonstrated to be markedly involved in the induction of ET^8^. BCL3, a member of the IκB family, is predominantly localized in the nucleus, interacts with and stabilizes p50 and p52 homodimers, and thus is known as a negative regulator of NFκB-mediated transcription^8, 41^. In RAW264.7 cells, an siRNA-mediated 60% reduction of BCL3 at the protein level was sufficient to significantly increase LPS-induced *Tnf* transcription^42^. In BMMCs, even a 100% reduction (as realized in the *Bcl3*−/− cells) did not cause enhanced production of *Tnf* and TNF-α in response to LPS. This suggests that in LPS-stimulated BMMCs other control mechanisms are in place to limit *Tnf* transcription and TNF-α production. Most likely, these control mechanisms are centred around the NFκB pathway.

Interestingly, in PMCs deficiency of BCL3 results in a marked increase in TNF-α production compared to WT PMCs, indicating considerable differences in composition and regulation of signalling pathways between various types of MCs. PMCs are connective tissue-type MCs and BMMCs are described to be more mucosal-type MCs. Nevertheless, because different MCs are known to exert great plasticity with respect to expression of proteases, it appears quite natural that their response pattern might also change depending on distinct stimuli. Thus, our investigation of BMMCs and PMCs and the differences found for the role of BCL3 in controlling LPS-induced TNF-α production suggests that different MC types possess distinct capabilities to regulate the expression of NFκB-dependent proinflammatory cytokines.

In summary, this study shows in BMMCs that transient H3K9me3 demethylation in the course of LPS stimulation is involved in the production of the proinflammatory cytokines IL-6 and TNF-α. Moreover, this process involves the cytoplasmic activation, nuclear translocation, and promoter binding of NFκB. These important signalling events are suppressed in LPS tolerant cells disabling the production of these proinflammatory cytokines. Intriguingly, IgE-mediated activation of the NFκB pathway is also suppressed in tolerant MCs, however, without affecting the production of IL-6 and TNF-α, which can be accounted for by IgE-mediated calcium mobilisation and activation of respective transcription factors (Fig. 7). Finally, the role of BCL3 was investigated, which has been assigned a function in ET in macrophages and the expression of which was upregulated in tolerant MCs. Interestingly, using WT and BCL3-deficient PMCs, BCL3 was found to repress TNF-α expression in both naive and tolerant cells, hence controlling ET in a quantitative rather than a qualitative manner.

## Methods

### Cell culture

BMMCs were generated according to procedures introduced by Razin *et al*.^43^. Bone marrow cells (2 × 10^6^ cells/ml) from 6 to 8 week old mice (129/Sv × C57BL/6) were cultured (37 °C, 5% CO_2_) as a single cell suspension in culture medium (RPMI 1640 medium containing 17.5% FCS, 1% X63Ag8–653-conditioned medium (as a source of IL-3^44^), 2 mM L-glutamine, 1 × 10^−5^ M 2-mercaptoethanol, 100 units/ml penicillin and 0,1 mg/ml streptomycin). At weekly intervals, the non-adherent cells were reseeded at 1 × 10^6^ cells/ml in fresh culture medium. By 4–6 weeks in culture, greater than 99% of the cells were KIT- and FcεRI-positive as evaluated by phycoerythrin (PE)-conjugated α-KIT antibodies (clone ACK45, BD Biosciences, San Jose, CA, USA) and FITC-conjugated α-FcεRIα antibodies (clone MAR1, eBioscience, San Diego, CA, USA), respectively. PMCs were collected from the same mice by a peritoneal lavage with 3 ml sterile phosphate-buffered saline (PBS)^45^. Cells were pelleted and resuspended in 2 ml sterile Gey’s balanced salt solution to lyse red blood cells. After addition of FCS and centrifugation, the cells were cultivated for 3–4 weeks in culture medium (see above) containing 1% CHO-SCF-conditioned medium (as a source of SCF)^34, 45^. C57BL/6 WT and *Bcl3*−/− BMMCs and PMCs were obtained from C57BL/6 WT and *Bcl3*−/− (*Bcl3* ^*tm1Rsch*^) mice^46^ and were differentiated *in vitro* using the same protocols. Experiments were performed in accordance with German legislation governing animal studies and following the Principles of Laboratory

Animal Care. Mice are held in the Institute of Laboratory Animal Science, Medical Faculty of RWTH Aachen University. The Institute holds a licence for husbandry and breeding of laboratory animals from the Veterinary Office of the Städteregion Aachen (Administrative District). The Institute follows a Quality Management System, which is certified according to DIN ISO 9001/2008. Every step in this project involving mice was reviewed by the animal welfare officer. All experiments were approved by the Landesamt für Natur, Umwelt und Verbraucherschutz NRW (LANUV), Recklinghausen (AZ 84-02.04.2016.A496).

### Reagents

R-form LPS from *Salmonella minnesota* (R595) was extracted and purified as described^47, 48^ and was a gift from M. Freudenberg and C. Galanos (MPI for Immunobiology and Epigenetics, Freiburg, Germany). Recombinant murine IL-33 (ALX-522-101-3010) was acquired from Enzo Life Sciences GmbH (Lörrach, Germany). The synthetic lipopeptide FSL-1 was obtained from Echaz Microcollections (Tübingen, Germany). DNP-HSA containing 30–40 moles DNP per mole albumin and monoclonal IgE with specificity for DNP (clone SPE-7) were purchased from Sigma (Deisenhofen, Germany). DMSO was obtained from Carl Roth GmbH & Co (Karlsruhe, Germany). The IKK-β inhibitor IV (CAS-507475-17-4) and the TAK1 inhibitor (5Z)−7-Oxozeaenol (CAS-66018-38-0) were purchased from Merck Millipore (Billerica, MA, USA). The Calcineurin inhibitor Cyclosporin A (BML-A195) was obtained from Enzo Life Sciences GmbH (Lörrach, Germany) and the Calcineurin/NFAT inhibitor INCA-6 (CAS-3519-82-2) was purchased from Tocris Bioscience (Bristol, UK). The lysine-specific demethylase inhibitor Pargyline (CAS-306-07-0) was obtained from Cayman Chemical Company (Ann-Arbor, MI, USA) and the lysine-specific demethylase inhibitor TCP was purchased from Stem Cell Technologies Germany GmbH (Köln, Germany).

### Induction of ET and stimulation of BMMCs and PMC

To induce ET, cells were incubated in cell culture medium with 300 ng/ml R-form LPS for 24 h (37 °C, 5% CO_2_). This concentration of LPS can be considered as low-to-medium dose with respect to MC stimulation^14^. Afterwards the cells were washed with PBS and resuspended in RPMI/0.1% BSA. Cells were adapted to 37 °C for 30 min and then stimulated with R-form LPS (1 µg/ml), FSL-1 (1 µg/ml), and IL-33 (2 ng/ml) for various time points. For stimulation with the antigen DNP-HSA (20 ng/ml), the cells were always preloaded with IgE (0.15 µg/ml) for 16 h.

### RNA preparation and quantitative RT-PCR

Total RNA from 4 × 10^6^ cells was extracted using the RNeasy Plus Mini Kit (Qiagen, Hilden, Germany) according to the manufacturer’s instructions. RNA (1 µg) was reverse transcribed using random hexamers (Roche, Basel, Switzerland) and Omniscript RT Kit (Qiagen, Hilden, Germany) according to the manufacturer’s instructions. qPCR was accomplished on a Rotorgene (Qiagen, Hilden, Germany) by using SensiMix SYBR green No-Rox Kit reaction mix (Bioline GmbH, Luckenwalde, Germany). Expression was normalized to the housekeeper gene *Gapdh*. The relative expression ratio taking into account the primer efficiencies was determined according to the publication by Pfaffl^49^. Primers: *Gapdh* (fwd: ACT CAA GAT TGT CAG CAA TGC A, rev: TGG TCA TGA GCC CTT CCA CAA; eff.: 1.9483), *Bcl3* (fwd: CCG GAG GCC CTT TAC TAC CA, rev: GGA GTA GGG GTG AGT AGG CAG; eff.: 2.27198), *Il6* (fwd: TCC AGT TGC CTT CTT GGG AC, rev: GTG TAA TTA AGC CTC CGA CTT G; eff.: 2.00934), *Tnf* (fwd: AGC ACA GAA AGC ATG ATC CGC, rev: TGC CAC AAG CAG GAA TGA GAA G; eff.: 2.1854).

### Cytokine ELISAs

Mouse IL-6 ELISAs (BD Pharmingen, Heidelberg, Germany) and TNF-α ELISAs (R&D Systems, Wiesbaden- Nordenstadt, Germany) were carried out according to the manufacturer’s instructions. Levels of cytokine production were measured in supernatants.

### Chromatin immunoprecipitation (ChIP)

ChIP was performed according to a modified Q_2_-ChIP protocol that was originally developed by the laboratory of Phillipe Collas (Oslo, Norway) and described in detail by Ullius *et al*.^50^. Antibodies for ChIPs were purchased from Abcam (Cambridge, UK). Antibodies: α-H3K4me3 (rabbit polyclonal, ab8580, #GR37544-1), α-H3K9me3 (rabbit polyclonal, ab8898, #GR22415-1), α-NFκB p105/p50 (rabbit polyclonal, ab7971, #GR40002-2), α-NFκB p65 (rabbit polyclonal, ab7970, #GR23347-2), α-histone H3 (rabbit polyclonal, ab1791, #924831). ChIP primers were obtained from Sigma-Aldrich (St. Louis, MO, USA). Primers: *Il6*-promoter (fwd: ACCCCCACCCTCCAACAAAGATTTT, rev: AGCGGTTTCTGGAATTGACTATCGTTC), *Il6*-body (fwd: TGGTCTTCTGGAGTACCATAGCTACCT, rev: GCTACCCTGAGGTGCCGGGT), *Tnf*-promoter (fwd: AGGTGTAGGGCCACTACCGCT, rev: TGTCCTCGCTGAGGGAGCTTCTG), *Tnf*-body (fwd: GGCTCAGCCACTCCAGCTGC, rev: GGGCCACCCGGGACCTCATA). With respect to specificity, fold change of p50 and p65 ChIP enrichment was calculated relatively to input control and normal rabbit IgG was subtracted as background control.

### Western blotting

Cells were pelleted and solubilized with 0.1% Na-deoxycholate and 0.5% NP-40 in phosphorylation solubilisation buffer (PSB) at 4 °C^51^. Whole-cell lysates were subjected to SDS-PAGE and Western blot analysis. The Western blot antibodies anti-BCL3 (rabbit polyclonal, clone C-14, sc-185), anti-GAPDH (mouse monoclonal, clone 6C5, sc-32233), anti-Lamin A/C (goat polyclonal, clone N-18, sc-6215), and anti-p38 (rabbit polyclonal, clone C-20, sc-535) were purchased from Santa Cruz Biotechnology (Dallas, TX, USA). The antibodies anti-P-p38 (anti-phosphoThr180/phosphoTyr182, mouse monoclonal, clone 28B10, #9216), anti-P-AKT (anti-phosphoSer473, mouse monoclonal, clone 587F11, #4051), anti-TAK1 (rabbit monoclonal, clone D94D7, #5206), anti-P-IκBα (anti-phosphoSer32, mouse monoclonal, clone 14D4, #2859), anti-NFκB p65 (rabbit monoclonal, clone C22B4, #4764), anti-P-IKK-α/-β (anti-phosphoSer176/180, rabbit monoclonal, clone 16A6, #2697), and anti-IκBα (rabbit polyclonal, #9242) were obtained from Cell Signaling Technologies (Danvers, MA, USA) and the antibody anti-p85 (rabbit polyclonal, #06-195) was acquired from Millipore (Billerica, MA, USA). The antibody anti-Tubulin (mouse monoclonal, clone B512, #T5168) was purchased from Sigma-Aldrich (St. Louis, MO, USA) and the antibody anti-NFκB p105/p50 (rabbit polyclonal, ab7971, #GR40002-2) from Abcam (Cambridge, UK).

### Cytoplasm-nucleus fractionation

Cytoplasm-nucleus fractionation was performed with the Nuclear Extract Kit (#40010) from Active Motif (Carlsbad, CA, USA) according to modified manufacturer’s instructions. Cells (20 × 10^6^/ml) were collected at 200 × g in a pre-cooled (4 °C) centrifuge for 5 min and the supernatant was discarded. For collection of the cytoplasmic fraction pellets were resuspended in Hypotonic Buffer (1x) containing protease and phosphatase inhibitors for 15 min on ice. After adding the Detergent and vortex for 10 sec at highest setting, the cells were centrifuged for 30 sec at 14.000 × g at 4 °C. The supernatant containing the cytoplasmic fraction was transferred into a pre-cooled reaction tube. To collect the nuclear fraction the supernatant was thoroughly removed, the pellet was washed with PBS (1x) and immediately lysed with pre-heated 1.25 × Laemmli-buffer at 95 °C for 10 min. Subsequently, cytosolic and nuclear lysates were sonicated at 4 °C for 20 min using a Branson Digital Sonifier (model 450-D; G. Heinemann, Schwäbisch Gmünd, Germany); conditions: amplitude, 10%; interval, 30 sec). After sonication, the samples were subjected to SDS-PAGE and Western blot analysis.

### Statistical analysis

Data were generated from independent experiments and analysed for homoscedasticity and Gaussian distribution. P-values were calculated by the unpaired two-tailed Student’s *t*-test or two-sided ANOVA with Tukey HSD or Dunnet’s post-hoc test and were adjusted for multiple comparisons by false discovery rate (FDR). All statistical analysis was performed using JMP version 10 (SAS, Cary NC, USA). Figures represent means and SD of n independent experiments (with n indicated in the respective figure legends) and were performed using GraphPad Prism. P-values of * < 0.05, ** < 0.01, and *** < 0.001 were considered statistically significant. Values higher than a p-value of 0.05 were regarded as not significant (n.s.).

## Electronic supplementary material

Supplementary information 1
