# Supplementary material for: Endotoxin tolerance in mast cells, its consequences for IgE-mediated signalling, and the effects of BCL3 deficiency
Source: Sci Rep. 2017 Jul 3;7:4534. doi: 10.1038/s41598-017-04890-4 (PMC5495797; doi:10.1038/s41598-017-04890-4)
Supplement: Supplementary file 1 — Supplementary information 1 [file 41598_2017_4890_MOESM1_ESM.doc]

Supplementary information

Endotoxin tolerance in mast cells, its consequences for IgE-mediated signalling, and the effects of BCL3 deficiency

Magdalena Poplutz^1^, Maryna Levikova^2^, Juliane Lüscher-Firzlaff^2^, Marina Lesina^3^, Hana Algül^3^, Bernhard Lüscher^2^, and Michael Huber^1^*

^1^ Institute of Biochemistry and Molecular Immunology, Medical School, RWTH Aachen University, Aachen, Germany

^2^ Institute of Biochemistry and Molecular Biology, Medical School, RWTH Aachen University, Aachen, Germany

^3^ Molecular Gastroenterology, Medical Clinic II, University Hospital Klinikum Rechts der Isar, TU Munich, Munich, Germany

^1^ Corresponding author: Dr. Michael Huber (PhD)

Institute of Biochemistry and Molecular Immunology

RWTH Aachen University

Pauwelsstr. 30, 52074 Aachen, Germany

Tel: ++49-241-8088830; Fax: ++49-241-8082428

E-mail: [mhuber@ukaachen.de](mailto:mhuber@ukaachen.de)

Supplementary Figure S1

FACS analysis of WT and *Bcl3-/-* BMMCs.


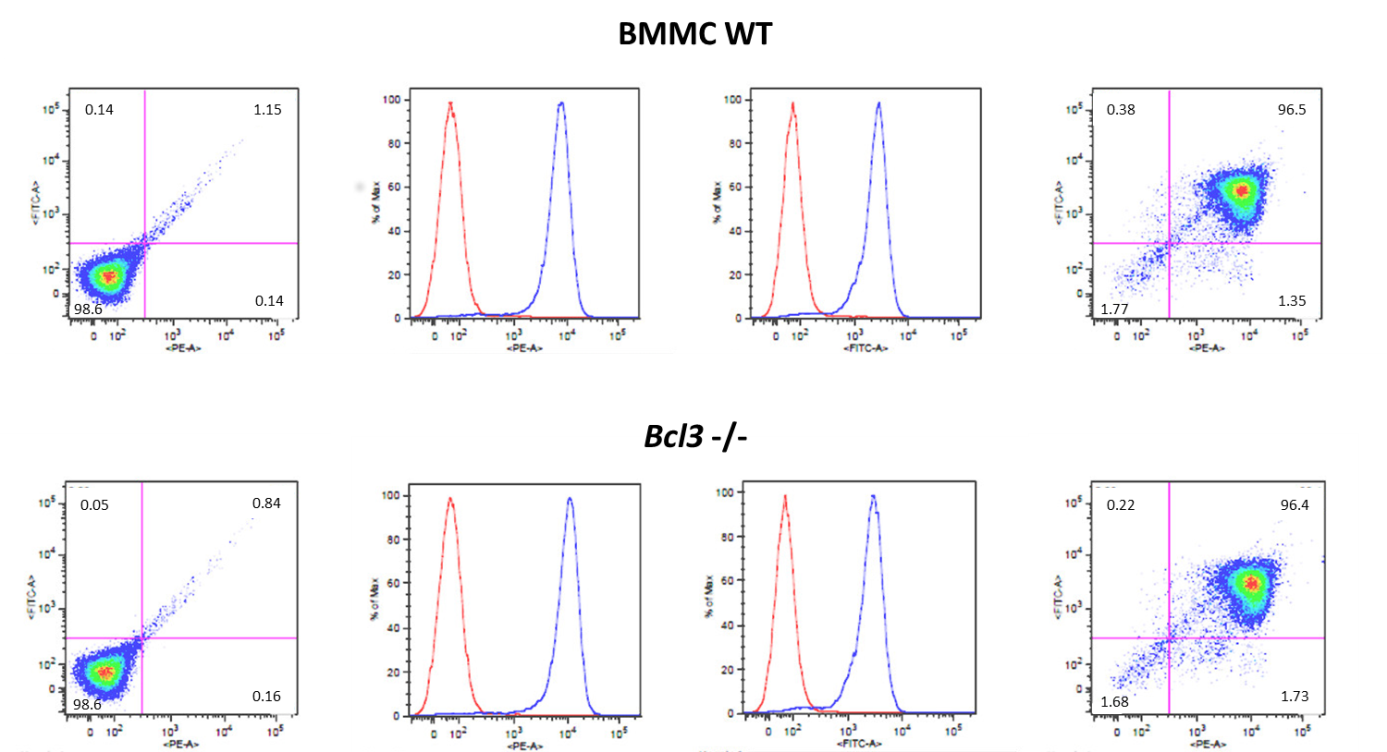


WT and *Bcl3*-/- BMMCs were analysed after 4-6 weeks in culture via FACS analysis for surface expression of FcεRI (FITC) and KIT (PE). Red histograms show autofluorescence, blue histograms FcεRI- and KIT-specific signals, respectively. Dot-blots show unstained (left panels) and double-stained WT and *Bcl3-/-* BMMCs (right panels). Cells were considered as ready-to-use when they displayed at least 95 % positivity for both receptors. Data shown are representative of n > 10 independently differentiated BMMC cultures.
